# Acceptability, simplicity, and relevance of the new human papillomavirus/DNA test among 35-year-old ever-married women in a district of Sri Lanka: focus group discussions

**DOI:** 10.1186/s12905-022-01712-2

**Published:** 2022-04-25

**Authors:** K. C. Perera, K. N. Mapitigama, T. C. Abeysena

**Affiliations:** 1 The Directorate of Non-Communicable Diseases, Ministry of Health, Colombo, Sri Lanka; 2 Family Health Bureau, Colombo, Sri Lanka; 3 Faculty of Medicine, University of Kelaniya, Gampaha, Sri Lanka

**Keywords:** Cervical cancer screening, SriLanka, HPV/DNA test, Relevance, Simplicity, Acceptability

## Abstract

**Background:**

Two major drawbacks of the present cervical cancer screening programme in Sri Lanka are, the suboptimal sensitivity of the pap smear and the low coverage. The sensitivity of the HPV/DNA screening test is high. The objective of the study was to explore the acceptability relevance and simplicity of the new HPV/DNA screening implementation among ever-married women in a district of Sri Lanka.

**Methods:**

Focus group discussions (FGD) (n = 3) in the public health divisions of the Kalutara district were used to collect data during December 2018. The study population comprised of ever-married women 35 years old, who, carried out an HPV/DNA test at a community Well Woman Clinics (WWCs) (n = 89). A list of WWCs was prepared according to an alphabetical order under urban, rural, and estate sector categories and allocated a number. One WWC was selected from each sector randomly for the three FGDs representing the estate, rural, and urban clinics. A convenient sampling technique was used to select participants for each FGD (n = 8). The information collected at each interview was summarized at the end of each interview. The analysis was done with manual content.

**Results:**

Most of the participants were Sinhalese (n = 17, 70.9%), Buddhist (n = 18, n = 75%), and non-working (n = 18, n = 75%). The community awareness of HPV/DNA screening and field staff performance were highly appreciated by most of the participants. Most were aware of the high sensitivity of the HPV/DNA test, therefore the early detection rate of cervical cancer precursors is high. Most of the participants expressed the HPV/DNA test as a convenient and neutral test. Most were mentioned the necessity of repeated clinic visits for the pap test and colposcopy in HPV/DNA screened positive follow-up but there was marked acceptability (n = 23, 95.8%) for HPV/DNA test.

**Conclusions:**

Acceptability of the new HPV/DNA screening test was high. Most of the participants perceived the HPV/DNA test to be simple and also relevant. Therefore, the HPV/DNA screening test can be recommended to be incorporated into the National Cervical Cancer Screening Programme as its suitability was well explored in the Sri Lankan setting.

**Supplementary Information:**

The online version contains supplementary material available at 10.1186/s12905-022-01712-2.

## Background

Cervical cancers are virtually associated with Human papillomavirus infection (HPV). Human papillomavirus infection is exclusively sexually transmitted. Nearly 60 different types of HPV serotypes are known to infect the human genital tract including the cervix uteri [1]. Some oncogenic serotypes are classified as "high risk" (16, 18, 31, 33, 35, 39, 45, 51, 52, 56, 58, and 59) for cervical cancer [2]. Compared to other carcinogenic serotypes of HPV infection, serotypes 16 and 18 have 190.3 times increased risk of developing cervical cancers [3].

Persistent carcinogenic HPV infection may progress into cervical precancerous lesions (Cervical Intraepithelial Neoplasia) within 10–20 years. The mean age of marriage for Sri Lankan females is 23.9 years [4]. Therefore the target age cohort for the National Cervical Cancer Screening programme in Sri Lanka was fixed at the age of 35 years (23.9 + 10 ~ 35 years) with the assumption of coitarche occurred only after marriage among Sri Lankan females.

Cofactors favouring HPV infection are not well defined and the following risk factors probably play a role; type of the HPV and its oncogenicity, commencement of sexual activity at an early age, multiple sexual partners, a partner who participates in high-risk sexual activities, immune suppression acquired or primary, poor socio-economic status-poor hygiene and connection with other sexually transmitted agents. Co-factors that precipitate HPV infection to persist are; high parity, tobacco smoking, poor nutritional status, prolonged usage of oral contraceptive pills (a slight increase in risk is observed with usage for ≥ 5 years, whereas usage for ≥ 10 years shows increased risk) [5].

Cervical cancer is one of the few cancers, where a precursor stage (pre-cancer) lasts decades before becoming invasive cancer giving an ample opportunity for early detection and timely treatment. Since these lesions precede cancer by decades, cervical cancer is therefore preventable. Moreover, if cervical cancer is detected early and treated appropriately, still it can be cured [5].

In 1998, Sri Lanka took an initiative to include screening for cervical cancer with conventional Papanicolaou (pap) smear in Well Woman Clinics (WWCs). However, even after 20 years of cervical cancer screening with conventional pap cytology, there was no marked reduction in incidence, morbidity, and mortality rates of cervical cancer in Sri Lanka [6]. Two major drawbacks of the present cervical cancer screening programme are suboptimal sensitivity (53%) of the pap smear [7] to detect Cervical Intraepithelial Neoplasia (CIN) and low coverage (53.3%) [8] of the programme. Therefore, it needs to pay urgent attention to reviewing the National Cervical Cancer Screening programme in Sri Lanka.

HPV/DNA screening methods are based on the detection of high-risk HPV/DNA in vaginal or cervical smears [9] and it's already practised in some developed countries [5]. There are many advantages of HPV/DNA testing as a screening test compared with a visual inspection of the cervix or cytology-based screening, these include; higher sensitivity, identification of the earliest cervical cancer precursor (HPV virus antigen), and lengthy screening intervals [10]. Besides, the interpretation of the test is objective and doesn't have the inherent subjectivity of visual screening methods or cytological assessment [11]. The average sensitivity and specificity of the HPV/DNA test in the 30–39 age cohort of women are 89% and 90% respectively [12].

Some randomized controlled studies have proven that screening women between 30 and 59 years, even by a single round of HPV/DNA antigen detection testing can significantly bring down the incidence of advanced cervical cancer and reduce cervical cancer mortality by 50% [13–15]. HPV/DNA screening is used in combination with cytology (co-test), as a triaging test for borderline cytology (Atypical Squamous Cells of Undetermined Significance {ASCUS} cytology with HPV/DNA test triage) or as a primary screening method followed by cytology i.e. HPV/DNA test with cytology triage. In primary screening, all HPV/DNA screen-positive women with ≥ ASCUS cytology results are only referred for colposcopy, therefore a marked reduction (40–50%) in the number of referrals for colposcopy can be expected [16].

Hence, the present National Cervical Cancer screening programme in Sri Lanka needs to be reviewed with special attention. The objective of the study was to explore the acceptability, relevance, and simplicity of new HPV/DNA screening implementation as a primary cervical cancer screening method among 35 -year old ever-married women in a district of Sri Lanka.

## Methods

### Study design and setting

A cross-sectional qualitative study design was used. Three FGD sessions were conducted in the public health administrative division called Medical Officer of Health (MOH) areas of Kalutara district in December 2018. The number of MOH areas in the Kalutara district was 15, while the total number of WWCs located was 89. The Medical officer of Health areas are further divided into Public Health Midwife (PHM) areas (n = 413). Public Health Midwife areas drained to each WWC were obtained from each MOH office in the Kalutara district.

### Study population

The study population comprised ever-married women 35 years of age, in MOH areas of Kalutara district, who had undergone HPV/DNA test at community WWCs in Kalutara district.

### Theoretical framework

**Fig. 1:**
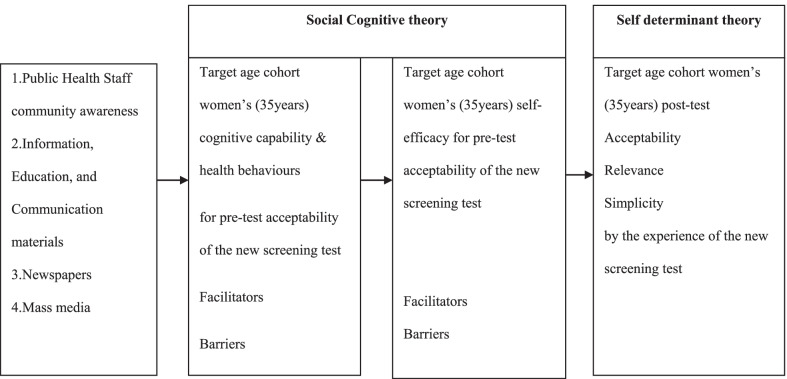
Conceptual framework of the FGDs

A conceptual framework (Fig. 1) was developed based on the literature [17, 18] to conduct FGDs. Using this conceptual framework, a moderator guide/FGD guide (Additional file 1) to facilitate the FGD was developed for this study with the assistance of experts. A moderator guide was reviewed by a panel of experts with a few preventive Specialists, and Medical Officer to determine the correct question. The key component of the conceptual framework was six comprehensive questions as stated below;

- What do you know about cervical cancer screening?
- What do you think about the present cervical cancer screening method in Sri Lanka?
- What are the weaknesses of the present cervical cancer screening method in Sri Lanka?
- What do you think about the new HPV/DNA screening test as a cervical cancer screening method in Sri Lanka?
- Was the given summary appropriate?
- Did you have any more things to tell?

Questions were open-ended and flexible but were focused on the research topic. Acceptability of the screening test mainly depends on the community's knowledge and attitudes about the screening test [18].

### Recruitment and consent

The type of population (e.g. urban, rural, and estate) in each PHM area was obtained from the Reproductive Health Management Information System, Family Health Bureau. Well Woman Clinics in the Kalutara district were categorized into estate, rural and urban clinics according to the type of population attending the clinic. A list of clinics was prepared according to the alphabetical order of the clinic name and allocated a number. Three clinics were randomly selected using a lot method from the prepared list for three FGDs with estate, rural and urban participants, who had undergone HPV/DNA tests at the selected WWC clinic.

Eight participants were selected four weeks before the conduction of FGDs by a convenient sampling technique from the selected WWCs for each FGD session. Participants were provided information on the venue and time for the discussion. Before data collection informed written consent was obtained from the selected participants by signing a consent sheet. Participants’ profiles were recorded.

### Data collection

The Principal Investigator, with advanced training in conducting FGDs, was the moderator. One science graduate was recruited as an assistant moderator to give background support by setting up the interview room, taking notes throughout the discussion, monitoring recording equipment, asking questions if invited and debriefing with the moderator at the end of each discussion[19]. Non-verbal messages were taken into note. All FGDs were audio-recorded following the verbal consent of participants.

The Moderator bias was reduced by remaining neutral during an interview as much as possible [20]. The Principal Investigator (PI) was unable to appoint an adequately trained suitable moderator due to financial constraints, therefore bias may have been introduced with participants on the knowledge of the Principal Investigator’s profession. To ensure respondent validation, information collected at each interview was summarized at the end of each interview.

### Data analysis

Three focus group discussion sessions were carried out. The first session was conducted as a pilot test and there were no significant alterations, therefore it was considered for the analysis. Audiotapes were transcribed verbatim by the PI on the day of an interview. Once the verbal data was collected and typed, repeated answers and re-current trends in raw data were marked and the most common answers then became the major results of the study [21]. Both audio and verbal transcripts were compared to ensure comprehensibility and prepared descriptive codes by the PI with all emerging information. Descriptive codes were modified to identify broad themes and categories. The analysis was done with manual content.

This procedure was continued until all discussions were conducted. These steps were followed up in all three FGDs and an amalgamated list of codes was developed under each broad theme [22]. Finally, the summary results were prepared.

## Results

Thirty-five years age cohort of ever-married women (n = 24) participated in the study. Most of the participants engaged in FGDs were Sinhalese (70.9%, n = 17), Buddhist (75%, n = 18),and Non-working (75%, n = 18).Out of the total participants, 20.8% (n = 5) had not completed years of school education beyond the 5th grade level and another 12.5% (n = 3) of the participants remained at the 6–11th grade level of education. The majority (62.5%, n = 15) were educated up to the level of Ordinary Level (O/L) passed education. Out of the participants, 20.8% (n = 5) were done Advanced Level (A/L) passed education. Some of the participants (16.7%, n = 4) were done a degree and above-passed education (Table 1).

**Table 1:** Participants’ profile of FGDs

Characteristics of participant	Number (n = 24)	Percentage %
Race		
Sinhalese Tamil Muslim	17 01 06	70.9 4.1 25.0
Religion		
Buddhist Catholic Muslim	18 03 03	75.0 12.5 12.5
Education level		
No schooling Grade 1–5 Grade 6–11 O/L passed A/L passed Degree and above	01 04 03 07 05 04	4.1 16.7 12.5 29.2 20.8 16.7
Occupational status		
Working Non-working	06 18	25.0 75.0

Following three FGD sessions, altogether six major themes were identified to express the acceptability, relevance, and simplicity of the new HPV/DNA screening test implementation and screen positive follow-up (Table 2). The results of the study component are presented under the following themes according to the knowledge, attitudes, and perceptions of participants of the relevant emerged theme.

**Table 2:** Main themes and codes emerged following FGDs among 35-year-old ever- married women in the Kalutara district

Main themes	Main descriptive codes
Cervical cancer situation in Sri Lanka	Cases, Treatment, Deaths
Human Papilloma Virus infection and cervical cancer	Carcinogenic effect, Cancer precursor, Sexually transmitted, Persistent infection
Pap test and HPV/DNA test as cervical cancer screening methods in Sri Lanka	Early detection, Pap smear test, Sensitivity, Accurate, Reporting delay, Screening interval
HPV/DNA specimen collection and screening implementation	Convenient, Neutral, Community awareness, Field staff, Clinic facilities, Easy, Clinic staff, Quick test, Environment, Quick report
HPV/DNA screening positive follow-up with pap test and colposcopy	Repeated clinic visits, Pap test, Colposcopy, Advance technique
Challenges associated with HPV/DNA screening	Hurting oneself, Administering the test correctly

(i) Cervical cancer situation in SriLanka

Most study participants claimed the cervical cancer incidence was still high in Sri Lanka. A participant particularly paid attention to some preventive measures for cervical cancers. This was expressed in this way:

"I got to know that in contrast to the vigorous preventive measures… including vaccination against HPV infection, screening with pap test, still annually a high number of new cervical cancer cases are reported…markedly" (Participant, urban sector).

Few participants stated that cervical cancer is the second most commonest female cancer in Sri Lanka according to the incidence rate. A participant expressed that cervical cancer ranks as the third commonest cancer according to the age-specific incidence rate among 15–44 years, women as follows:

"I recently read one newspaper article, it says…cervical cancer is the second commonest female cancer in Sri Lanka, but among 15-44 years women it ranks as third…" (Participant, urban sector).

Most of the study participants indicated the treatment of cervical cancer was costly. One of the participants stated her experience as follows:

"My relative sister is suffering from cervical cancer…it was a late diagnosis…She had undergone an abdominal hysterectomy…Now the biggest problem is she is on the course of chemotherapy…., She has to spend big money on some drugs…I don't know the exact price…" (Participant, rural sector).

The lack of treatment facilities for cervical cancer in the rural sector of Sri Lanka was an issue mentioned by a few participants. A participant had this to say:

"One of my known cervical cancer patients has to travel daily to Colombo for chemotherapy, lack of treatment facilities in the rural sector is a big issue. And the cost for transport, transport difficulties, unable to occupy and carry out routine work is an extra burden for the patient…" (Participant, rural sector).

One expressed chemotherapy as the key challenge for a patient under treatment for cervical cancer by the following quote:

"It was such a worse experience than anything…, The biggest problems following the chemotherapy were loss of appetite, nausea, constipation, poor sleep, and lack of energy…[seems somebody has to look after her]. Nobody, she does her work slowly" (Participant, urban sector).

Some discussants mentioned that deaths due to cervical cancers are high in Sri Lanka. This was captured this way:

"I exactly can't remember the number of deaths due to cervical cancer in last year… but it says still it's high…" (Participant, estate sector).

Two study participants admitted that deaths due to cervical cancer remain stable for a long duration. The place to seek treatment is also related to the financial situation of the women. This was how one of the participants presented it:

"You can see clearly, irrespective of the development of treatment facilities…deaths due to cervical cancer remain stable for a long duration of years...access for treatment centres and treatment cost plays important role in this regard" (Participant, urban sector).

(ii) Human papillomavirus infection, and cervical cancer

Most of the participants gained knowledge on the effects of HPV infection and its association with cervical cancer following the community awareness sessions carried out in the district. A participant admitted in her words:

"I got to know effects related to HPV infection, carcinogenic and non-carcinogenic…all cervical cancers are virtually associated with…HPV infection" (Participant, rural sector).

From the views of the few discussants, they were the first time heard about the HPV virus and HPV infection as a cervical cancer precursor. A participant had this to say:

 "I never knew that there is a virus called HPV…Which has a cervical cancer carcinogenic effect…I heard that HPV infection as an initial cervical cancer stage…[precursor], Which could be detected by screening" (Participant, rural sector).

One indicated some serotypes of HPV are more carcinogenic than others and the contribution of HPV serotypes 16 and 18 to cervical cancer is high among all high-risk carcinogenic HPV serotypes and this was pointed out this way:

"There are highly carcinogenic virus types too… I have explained the variety of high-risk HPV serotypes, It was around 15…. I can't remember all but HPV types 16 and 18 are contributed to 70% of cervical cancer cases…" (Participant, urban sector).

Among participants, most stated the mode of transmission of HPV is exclusively sexual. One of the discussants captured it this way:

"I came to do screening test for HPV/DNA on the given appointment date…I've noticed in the clinic, informative posters were hanging on clinic walls…It says… HPV infection is exclusively sexually transmitted…" (Participant, rural sector).

From the views of some study participants, primary preventive methods for cervicovaginal HPV infection are readily available in Sri Lanka. Fortunately, their understanding of preventive measures is great. One said:

"There are some primary preventive methods for cervicovaginal HPV infection in Sri Lanka, condoms during a sexual relationship, and recently introduced vaccines…" (Participant, estate sector).

Few discussants noted persistent HPV infection may lead to cervical cancer. One of the participants remarks:

"It says high-risk carcinogenic HPV infection may progress into cervical cancer after about 10 to 15 years [persistent infection]…" (Participant, urban sector).

(iii) Pap test and HPV/DNA test as a cervical cancer screening method

Most of the study participants stated that the pap test is a National Cervical Cancer screening method in Sri Lanka and they knew that it can be done at either the MOH office level or hospital WWCs. This was captured this way:

"My one of the closest friends had some clinical signs and symptoms…She had undergone repeated pap tests for cervical cancer screening as requested by the Gynecologist. First pap smear, she has done at a MOH area WWC…and repeated ones at hospital WWC" (Participant, rural sector).

For some, suboptimal sensitivity of the pap test was a priority. Therefore missing many cervical precancerous lesions is unavoidable. One of the participants expressed it this way:

"I recently went to do my first pap screening test, The biggest problem of the pap test is…missing lesions...[suboptimal sensitivity]. Many chances to miss cervical abnormalities by a pap test only…" (Participant, rural sector).

Few discussants mentioned they would get the pap report with a lag period time. This was presented this way:

"I was asked to attend WWC for a pap test in the middle of this year by the area PHM…I did it so “My pap report received only after 6wks…luckily it was normal… [No Intraepithelial Malignancy]". (Participant, estate sector).

One participant indicated that the routine screening interval of the pap test is once in every 5 years and shared her experience this way:

"My pap report was normal….nothing else to be done, only routine follow-up after 5 years…" (Participant, urban sector).

From the views of most of the participants HPV/DNA test is an advanced screening test to detect cervical precancerous lesions as its sensitivity is high. According to one participant:

"It is wonderful to have such an advanced screening test with high detection rate…[sensitivity] as cervical cancer is one of the commonest cancer among Sri Lankan females…" (Participant, rural sector).

Some participants relied more on the higher accuracy of the HPV/DNA test due to cobas 4800 machine screening report. One of the participants remarked:

"HPV/DNA screening test is a cobas 4800 machine screened test…[accurate]. Screening never depends on any individual. I can't explain the variability that might occur in screenings based on personals [cytology]…" (Participant, urban sector).

The HPV/DNA test detects the initial precursor before the cervical lesion development was, expressed by most of the participants. A participant described her experience this way:

"I was at a community awareness session in the district…It was explained that HPV is one of the earliest stages that can be identified as a cervical cancer precursor. HPV/DNA test detects the presence of HPV virus in the smear…" (Participant, rural sector).

Few participants greatly appreciated getting of such an advanced screening test free of charge in the Government sector to reduce the burden of cervical cancer in Sri Lanka. A participant shared her experience this way:

"It was such an opportunity to screen for cervical cancer free of charge...such an advanced test…I am not well aware of the exact cost of the test, but it says it is expensive…much more expensive than a pap test" (Participant, estate sector).

The advantage of getting a quick test report was mentioned by two participants and this was pointed out this way:

"After passing one week following the test…My area PHM informed me that my test report can be collected, I did it…" (Participant, rural sector).

iv. HPV/DNA test specimen collection procedure and screening implementation

Most of the participants perceived an HPV/DNA screening test as a convenient and neutral test by their experience after exposure to the specimen collection at WWCs. A participant shared her experience this way:

"I've undergone a pap test early in this year at a community WWC, It is very similar to the pap test…Specimen collection was done by using a broom-like device, instead of the wooden spatula in the pap test, and then putting it into a liquid container. Push the broom-like device against the bottom of the vial 10 times, forcing the bristles to bend apart. Swirl the broom vigorously after pushing…simple and convenient, I thought it is painful, but the specimen collection was without any pain[neutral]" (Participant, rural sector).

HPV/DNA test is perceived as an easy test by some participants. According to one participant:

"I was really afraid to undergo HPV/DNA screening, but It is an easy and comfortable test…" (Participant, estate sector).

Few discussants noted that the HPV/DNA test is a quick one. One said:

"…for me, It was such a quick test, but I exactly couldn't calculate the time spent on the procedure, may be around 15 min…" (Participant, urban sector).

Well-organized community awareness and field staff performance for the new HPV/DNA test implementation was highly appreciated by most of the participants. A participant admitted in her words:

"I got to know information regarding this new screening test from the community awareness programme held at our MOH area, and leaflets were distributed among participants there…Lectures and discussions were carried out. I've seen an informative poster in all three languages, containing important details regarding HPV infection…Participants asked questions, and cleared doubts…I am so lucky, I got an opportunity to clarify doubts from my area PHM…after I got selected for a free HPV/DNA test…She visited me twice to make me aware…to reduce the anxiety…" (Participant, estate sector).

Some were satisfied with the clinic facilities and clinic staff’s performance in implementing HPV/DNA screening test. One of the participants shared her experience this way:

"Unlike the pap test, I doubted how this new test is being carried out in our clinic setup… I have noticed the clinic appointment schedule is displayed on the wall, a few more specific items were available at the clinic…If you go to a clinic, in addition to normal WWC clinic items, you can see unopen test kits, special marker pens to write the identification numbers are also available there…" (Participant, rural sector).

Sharp bins to discard clinical wastes are available at clinic premises. The environment following a clinic session was exemplified by the following quote:

"That clinic day, I was the last person…and I noticed at the end of clinic session…all brushes, which were used to obtain specimen was disposed to a sharp bin, clinic environment was so clean and clear…" (Participant, estate sector).

xxii. HPV/DNA screen positive follow-up with pap test and colposcopy

Most discussants admitted the necessity of repeated clinic visits for a pap test and colposcopy in HPV/DNA screen positive follow-up as exemplified by the following two quotes:

"I was informed HPV/DNA screen positive results by MOH….I was called up at the MOH office…MOH personally handed over my report…[anxiety]. It was explained that all HPV/DNA screen positives are not associated with cervical cancer…only need to undergo a pap test within the next 6 weeks…to reassure"  

"if the pap test is negative,…then to repeat an HPV/DNA test after 1 year…When the pap test is positive,…Then to be subjected to a colposcopy within one month after the test[to exclude cervical lesion]. Colposcopy is the gold standard test to diagnose cervical lesions as I heard" (Participant, urban sector).

For some discussants, irrespective of the repeated clinic visits HPV/DNA screen positive follow-up is very useful. It significantly reduced the cervical cancer burden in Sri Lanka due to the early detection of cervical cancer precursors. This was presented this way:

"I would be happy to do all relevant follow-up tests…related to HPV/DNA test, despite I have to go for repeated clinic visits …as I would say it’s an advanced cervical cancer screening method, with a high detection rate of early stages of cervical cancer[precursor]. Therefore it reduces the burden of morbidity, treatment cost, and mortality of cervical cancer" (Participant, urban sector).

(vi) Challenges associated with HPV/DNA screening

All of the participants had seen the specimen collection brush only on the day of specimen collection in the WWC. One participant shared her experience as follows:

"I went to the WWC on the scheduled day, I was informed the specimen is collected by using a brush… the First time I've seen the brush at the clinic day only...It’s actually like a broom" (Participant, urban sector).

Fear of hurting themselves was the biggest barrier associated with the HPV/DNA screening. Further, some thought even HPV/DNA specimen collection is very painful. One of the participants presented it this way:

"I was really scared [hurting myself] of participating in HPV/DNA screening… as I thought the specimen collection is very painful, there is no pain, only a tingling sensation, nothing else…" (Participant, estate sector).

Some participants doubted whether this test is administered correctly by the health staff. One participant described her experience this way:

"We were shown some videos of HPV/DNA specimen collection during the community awareness sessions in the district, I was wondering how such a different screening test could be able to correctly administer by our staff suddenly…" (Participant, rural sector).

Prior conduction of well-organized staff training for HPV/DNA specimen collection and training was very useful to raise the community participation in the study. This was presented this way:

"I got to know that well-organized staff training under expertise was conducted at district level on HPV/DNA specimen collection and screening…" (Participant, urban sector).

Two participants pointed out that continuous monitoring and supervision of this new HPV/DNA screening implementation is essential to assure the standards. One of the discussants captured it this way:

"Staff may not adhere to screening norms…need continuous monitoring and supervision of HPV/DNA specimen collection and screening". (Participant, rural sector).

One said issues arising in test implementation must be discussed with expertise periodically as exemplified by the following quote:

"I know how difficult to implement a new test…many issues need to be sorted out…It is essential to conduct periodical reviews with expertise…" (Participant, urban sector).

There was marked acceptability among participants (n = 23, 95.8%) for HPV/DNA screening tests to be incorporated into the National Cervical Cancer Screening Programme in Sri Lank as a primary cervical cancer screening method, irrespective of all difficulties in repeated clinic visits for a pap test and colposcopy in the screen-positive follow-up.

## Discussion

Cervical cancer is the second most commonest female cancer in Sri Lanka [23]. Therefore, Sri Lanka took an initiative to include screening for cervical cancer with conventional Papanicolaou (pap) smear in the WWCs in 1998. After 20 years of the existence of the programme, in contrast to the vigorous preventive measures, there is no marked reduction in incidence, morbidity, and mortality of cervical cancer in Sri Lanka. Primary screening for cervical cancer is now transiting from the longstanding cervical cancer screening method, so-called pap screening to a new HPV/DNA screening test, which is more sensitive than pap cytology in the detection of CIN to reduce the burden of cervical cancer incidence in a country.

Based on the results of existing studies, many countries are recommending and implementing HPV/DNA testing-based screening programmes. Before implementing HPV/DNA screening test into a National Cervical Cancer Screening programme in a country, the need to understand factors, which will impact the acceptability of HPV/DNA screening test (e.g. knowledge, attitudes) by a woman is very crucial to ensure the smooth implementation of HPV/DNA test. The present study indicates the knowledge and attitudes of women related to the National Cervical Cancer Screening programme were adequate to decide on the new HPV/DNA test.

Mixed method research synthesis categorized relevant factors related to HPV/DNA primary screening for cervical cancer and described their influence on women's acceptability of HPV/DNA testing [24]. Results were shown that most factors associated with HPV/DNA test acceptability are included in the health belief model and/or theory of planned behaviour (e.g. knowledge, attitudes) but some other important factors are not encompassed by these theoretical frameworks (e.g. health behaviours, negative emotional reaction related to HPV/DNA testing).

The direction of influence of psychosocial factors on HPV/DNA test acceptability was formed based on 14 quantitative studies. These influences were such as; facilitators (e.g. HPV/DNA test benefits), barriers (e.g. lengthy screening intervals, difficulties in positive follow-up screening procedure), contradictory history (e.g. sexual history), and impact of HPV/DNA screening (e.g. high perceived severity of HPV infection). In the present study, community awareness was done before conducting the study on the benefits of HPV/DNA screening, which may have led to the higher acceptability of HPV/DNA test among women screened.

There was a marked elevation in the prevalence of cervicovaginal HPV infection from 2009 (3.3%) [3] to 2019 (6.2%) among the ever-married women population in Sri Lanka, which was a good influence on HPV/DNA screening. Only ever-married women were recruited for the present study as unmarried women are usually reluctant to mention their sexual history due to cultural stigma, which may lead to information bias.

As mentioned in some other studies challenges for the HPV/DNA screening were identified as difficulties of repeated clinic visits in HPV/DNA screen positive follow-up [24, 25]. Some participants claimed, that before the procedure they were reluctant about the new HPV/DNA screening test as they believed, it will hurt them and they were in doubt about whether this test is administered correctly by health staff as HPV/DNA test was introduced in the first time in the country. A similar pattern of challenges was reported in some other countries when HPV/DNA test was introduced as a cervical cancer screening method due to a lack of knowledge and experience [24–26].

This study was restricted to one district out of 25 districts in Sri Lanka due to logistic constraints. Women who had undergone HPV/DNA test were selected for FGDs as they only can give perceptions of acceptability, relevance, and simplicity of new HPV/DNA tests with their experience. The initial HPV/DNA test feasibility study was conducted in the Kalutara district, therefore FGDs were conducted among women who carried out an HPV/DNA test in the same Kalutara district. The population characteristics and the public health infrastructure of the district favoured the generalizability of the research findings to the whole country.

Comparison of screening results between vaginal and cervical (gold standard) methods of HPV/DNA specimen collection needs to be carried out to improve the coverage of cervical cancer screening programme as vaginal specimen collection doesn't require speculum insertion, therefore primary health care workers also can collect HPV/DNA specimen, while during their home visits.

Implementation of HPV/DNA screening in the National cervical cancer screening programme in Sri Lanka will improve the quality of the programme as the sensitivity of the HPV/DNA test is high. Therefore incidence, morbidity, and mortality of the cervical cancer burden will ultimately be reduced in the country.

## Conclusions and recommendations

The present study has revealed the high acceptability of the HPV/DNA screening test among 35 years age cohort of ever-married women and the relevance and simplicity of the test were high as perceived by them. Therefore, HPV/DNA screening test as a primary cervical cancer screening method is recommended to be incorporated into the National Cervical Cancer Screening Programme in Sri Lanka among 35 year age cohort of ever-married women as the prevalence of HPV infection and feasibility of new HPV/DNA screening implementation was well assessed in Sri Lankan setting.

## Supplementary Information

**Additional file 1.** A Moderator Guide for Focus Group Discussion.

## Data Availability

The datasets used for analysis in this study are available from the corresponding author on reasonable request. Data supporting research findings can be found.

## Abbreviations

HPV: Human Papillomavirus Infection
Pap: Papanicolaou smear
WWC: Well Woman Clinic
CIN: Cervical Intraepithelial Neoplasia
ASCUS: Atypical Squamous Cells of Undetermined Significance
FGD: Focus Group Discussions
MOH: Medical Officer of Health
PHM: Public Health Midwife
PI: Principal Investigator
O/L: Ordinary Level
A/L: Advanced Level
