# Supplementary material for: Acceptability, simplicity, and relevance of the new human papillomavirus/DNA test among 35-year-old ever-married women in a district of Sri Lanka: focus group discussions
Source: BMC Womens Health. 2022 Apr 25;22:131. doi: 10.1186/s12905-022-01712-2 (PMC9036817; doi:10.1186/s12905-022-01712-2)
Supplement: Supplementary file 1 — Additional file 1. A Moderator Guide for Focus Group Discussion. [file 12905_2022_1712_MOESM1_ESM.docx]

**Focus Group Discussion -Moderator Guide**

**Objectives of the Focus Group Discussion**

- To explore the acceptability of new HPV/DNA screening implementation among a 35year age cohort of ever-married women in a district of Sri Lanka.
- To explore the relevance of new HPV/DNA screening implementation among a 35year age cohort of ever-married women in a district of Sri Lanka.
- To explore the simplicity of new HPV/DNA screening implementation among a 35year age cohort of ever-married women in a district of Sri Lanka.

**Introduction**

Introduce the moderator and note-taker. Welcome the participant and show your gratitude for his/her participation

………………………………………………………………………………………………………………………………………………………………………………………………

………………………………………………………………………………………………

This interview is held to gather your views on the acceptability, relevance, and simplicity of new HPV/DNA screening implementation among a 35year age cohort of ever-married women in the Kalutara district. Communicate objectives to participants and provide information sheets to them.

“I would like to obtain your permission to take down notes, while during the discussion and record the session using an audio recorder to make sure none of your ideas or points is missed.

Participation in the discussion is a volunteer. Confidentiality of participants will be ensured by not including any identification details and information gathered will be kept confidential. Audiotapes will be erased after the completion of notes. The approximate time duration will be one hour.

Refreshment is available for all participants at the end of the discussion. Bathroom facilities are available at the…………………………you may go to the bathroom even during the middle of the discussion.

**Ground rules for the discussion**

- There is no right for the wrong answer to questions. Every ones’ ideas are important.
- This is an interactive session. All participants should contribute to the discussion. Do not wait till you are called up.
- Participants should feel free to disagree with each other.
- Let one person speak at a time.

**Questions for participants**

The facilitator should attempt to cover all listed questions. But it is not strictly necessary. Questions do not have to be asked in the stated order. Treat the questions as a means to initiate and continue the flow of conversation in the correct direction.

Initiate the conversation by asking for a round of self-introduction

1. What do you understand about methods of cervical cancer screening in Sri Lanka?

2. Is it important. If so why?

3. What do you think about cervical cancer screening with HPV/DNA test?

4. Do you think is it important. If so why?

5. What are existing problems with HPV/DNA screening?

6. What motivated you to participate in this HPV/DNA screening?

7. What are the strengths of the HPV/DNA screening test?

8. What are the weaknesses of the HPV/DNA screening test?

9. Do you think the HPV/DNA test is important to be incorporated into the National Cervical Cancer screening programme in Sri Lanka? If so why

10. What are your suggestions regarding the HPV/DNA screening test as a cervical cancer screening method in Sri Lanka?

11. Was this an appropriate summary?

12. Did you forget something?

If no more information is being produced, end the interview. Ask the note taker to summarize the data collected for participants. Ask participants, if all information has been stated and whether there is any more information to be added.

In the end, thank all participants for their valuable ideas and time. Provide refreshments.
